# Targeting cancer-related inflammation with non-steroidal anti-inflammatory drugs: Perspectives in pharmacogenomics

**DOI:** 10.3389/fphar.2022.1078766

**Published:** 2022-12-05

**Authors:** Hongjin Lai, Yi Liu, Juan Wu, Jie Cai, Hui Jie, Yuyang Xu, Senyi Deng

**Affiliations:** ^1^ Institute of Thoracic Oncology and Department of Thoracic Surgery, West China Hospital, Sichuan University, Chengdu, China; ^2^ West China School of Medicine, West China Hospital, Sichuan University, Chengdu, China; ^3^ Department of Outpatient, West China Hospital, Sichuan University, Chengdu, China

**Keywords:** cancer, inflammation, cyclooxygenase, non-steroidal anti-inflammatory drugs, pharmacogenomics

## Abstract

Inflammatory processes are essential for innate immunity and contribute to carcinogenesis in various malignancies, such as colorectal cancer, esophageal cancer and lung cancer. Pharmacotherapies targeting inflammation have the potential to reduce the risk of carcinogenesis and improve therapeutic efficacy of existing anti-cancer treatment. Non-steroidal anti-inflammatory drugs (NSAIDs), comprising a variety of structurally different chemicals that can inhibit cyclooxygenase (COX) enzymes and other COX-independent pathways, are originally used to treat inflammatory diseases, but their preventive and therapeutic potential for cancers have also attracted researchers’ attention. Pharmacogenomic variability, including distinct genetic characteristics among different patients, can significantly affect pharmacokinetics and effectiveness of NSAIDs, which might determine the preventive or therapeutic success for cancer patients. Hence, a more comprehensive understanding in pharmacogenomic characteristics of NSAIDs and cancer-related inflammation would provide new insights into this appealing strategy. In this review, the up-to-date advances in clinical and experimental researches targeting cancer-related inflammation with NSAIDs are presented, and the potential of pharmacogenomics are discussed as well.

## Introduction

As a fundamental innate immune response, inflammation is involved in tissue repair, defending against pathogens and other danger signals. Transient and well-organized inflammation is salutary while chronic inflammation has been proved to be related to the development of different malignancies (Grivennikov et al., 2010; Elinav et al., 2013; Greten and Grivennikov, 2019). Chronic inflammation can be evoked by both infectious and non-infectious processes of chronic injury or irritation, especially in organs exposed to the external environment (Ulrich et al., 2006). Besides, cancer-intrinsic and therapy-induced metabolic changes, cell stress and cell death are also important sources of cancer-associated inflammation (Hou et al., 2021). Chronic inflammation is regarded as an aberrantly prolonged immune response which results in epigenetic alterations that drive cancer initiation and progression, as well as the accumulation of growth factors that support the development of nascent cancer (Hou et al., 2021). Continuous production of various inflammatory molecules (cytokines, chemokines, prostaglandins, *etc.*) and recruitment of inflammatory cells within the tumor microenvironment (TME) promote tumor progression, metastasis and even therapy resistance (Greten and Grivennikov, 2019; Wang D. et al., 2021; Hou et al., 2021).

Preclinical and epidemiological evidences suggest that agents with anti-inflammatory effect, such as non-steroidal anti-inflammatory drugs (NSAIDs), have the potential to prevent or delay cancer initiation and improve therapeutic efficacy of cytotoxic agents, targeted agents and immune checkpoint inhibitors (Crusz and Balkwill, 2015). NSAIDs comprise a group of structurally diverse chemicals that can reduce the synthesis of prostaglandins by inhibiting the activity of the cyclooxygenase (COX) enzymes and other COX-independent pathways. The expression level of COX-2, an inducible isoform of the COX enzyme family, has been found elevated in breast cancer, prostate cancer, pancreatic cancer, lung cancer, bladder cancer and so on (Hashemi Goradel et al., 2019). Aspirin, one of the most classical NSAIDs, has been proved to be associated with decreased incidence and mortality of colorectal cancer (Li et al., 2015; Wang L. et al., 2021). Defined as pharmacological intervention to prevent or delay the process of carcinogenesis, cancer chemoprevention is now considered a practicable approach, especially with NSAIDs. In patients diagnosed with malignancies, concurrent use of NSAIDs with cytotoxic agents, targeted agents or immune checkpoint inhibitors seems to be hopeful as well (Edelman et al., 2008; Cortellini et al., 2020; Liu et al., 2020).

Despite the promising anti-cancer effects of NSAIDs, their treatment response varies among patients for many reasons, particularly because of inter-individual genetic differences of specific genes that are involved in drug metabolism or drug-induced signal transduction, and certain genetic variations have a significant impact on pharmacokinetics and effectiveness of specific drugs (Ulrich et al., 2006). Accordingly, the study of variations of genetic characteristics related to drug response is defined as pharmacogenomics (Roden et al., 2019). For instance, carriers of specific NF-κB variants might benefit from NSAIDs in cancer chemoprevention (Chang et al., 2009; Seufert et al., 2013). Therefore, taking account of relevant pharmacogenomic differences makes it possible to enhance the chemopreventive and therapeutic potential of NSAIDs in the treatment of malignancies.

In this article, we summarized general concept of inflammation and cancer development, and then highlighted advances in NSAID-targeted pro-cancer mechanisms involved in this process. The distinct results of clinical studies in chemoprevention and treatment of cancer with NSAIDs were presented and discussed as well. Furthermore, NSAID metabolism and its anti-cancer mechanisms, as well as related pharmacogenomic characteristics, were demonstrated. Factors affecting the effectiveness or risk of NSAIDs other than pharmacogenomic features were also mentioned.

## Inflammation and cancer development

Inflammation is a defensive response against infection and tissue injury, which can constrain the spread of pathogens or facilitate tissue repair. In the initiation of inflammation, pathogen-associated molecular patterns (PAMPs) expressed by pathogens or damage-associated molecular patterns (DAMPs) produced in sterile tissue injury or infection-related cell damage can be recognized by pattern recognition receptors (PRRs), which are generally expressed by innate immune cells, such as mast cells, tissue macrophages and dendritic cells (Zhao et al., 2021). Then these innate immune cells activate signal transduction pathways that promote the antimicrobial or proinflammatory functions, including secreting proinflammatory cytokines, chemokines and vasoactive amines (Zindel and Kubes, 2020). As a consequence, leukocytes and plasma proteins involved in innate immunity are recruited to sites of infection or tissue injury, where they start to eliminate microbes or cell debris and repair damaged tissue in a well-orchestrated way (Zindel and Kubes, 2020). Actually, when inflammatory cell recruitment reaches its peak, it is typically followed by clearance of these cells and the restoration of tissue homeostasis, and this process is known as resolution (Fullerton and Gilroy, 2016). However, if the proinflammatory stimulus is not eliminated during the acute phase of inflammation within several days or weeks, it will lead to incomplete or frustrated resolution and then develop into chronic inflammation, which has been proved to be associated with an increased risk for cancer (Grivennikov et al., 2010; Fullerton and Gilroy, 2016; Zhao et al., 2021).

**FIGURE 1:**
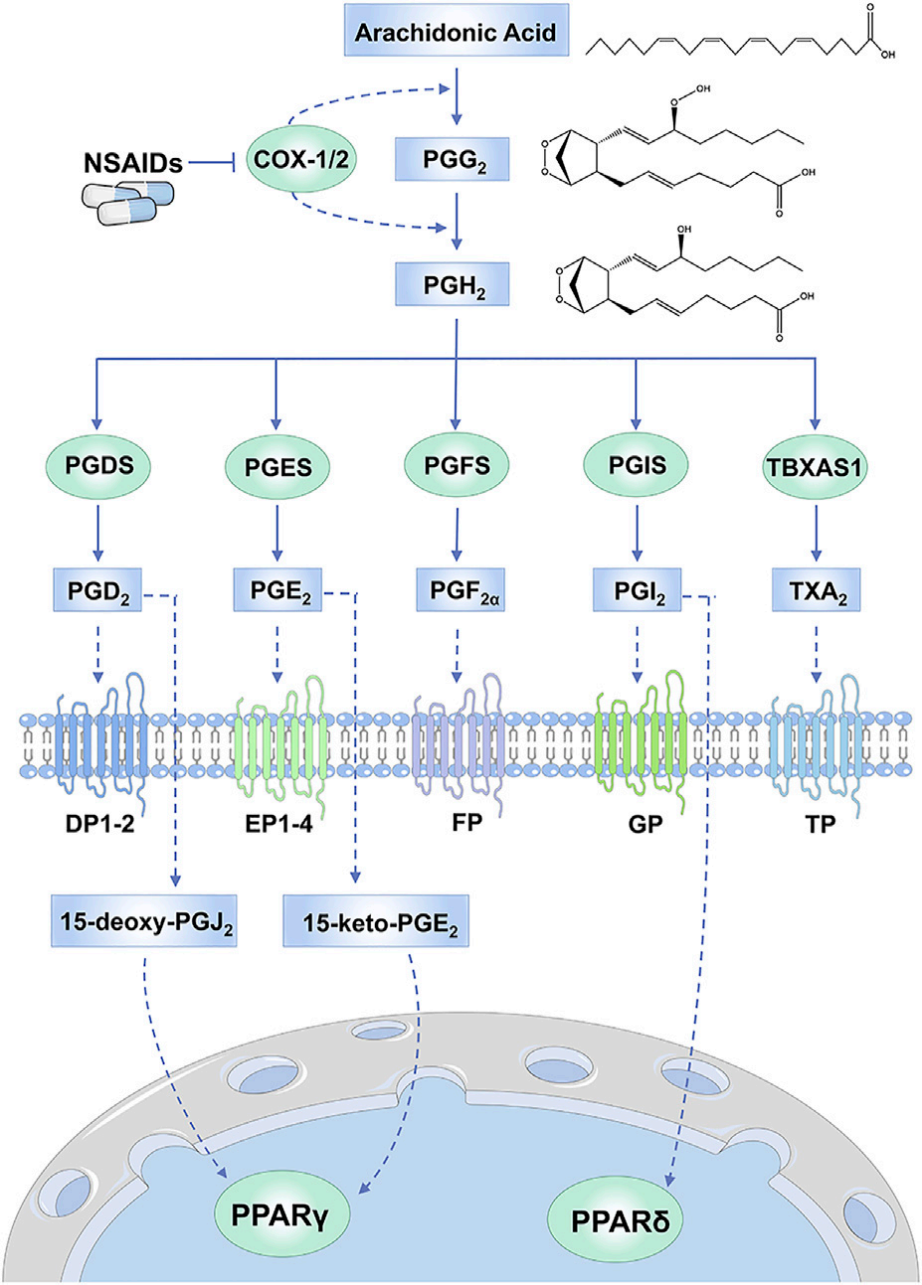
Synthetic and signal transduction pathways of prostaglandins. Arachidonic acid can be transformed into PGG_2_ and PGH_2_ *via* COX enzymes, which can be inhibited by NSAIDs. Then PGH_2_ is converted into various prostaglandins *via* specific synthases. Prostaglandins then exert their actions by activating receptors on cell membranes, including DP1-2, EP1-4, FP, IP and TP. Nuclear receptors such as PPARγ and PPARδ can also be activated by prostaglandins or their metabolites. Abbreviations: PGG_2_, prostaglandin G_2_; PGH_2_, prostaglandin H_2_; PGDS, PGD synthase; PGES, PGE synthase; PGFS, PGF synthase; PGIS, PGI synthase; TBXAS1, TXA synthase; peroxisome proliferator-activated receptor (PPAR).

During acute and chronic inflammation, the expression levels of proinflammatory molecules are upregulated, such as interleukin (IL)-1β, tumor necrosis factor (TNF)-α and interferon (IFN) γ, which are able to induce the synthesis of various eicosanoids, including prostaglandins (Aoki and Narumiya, 2012; Wang B. et al., 2021). Prostaglandins (PGs, including PGD_2_, PGE_2_, PGF_2α_, PGI_2_ and thromboxane A_2_) are synthesized from arachidonic acid by cyclooxygenase (COX), whose Human Genome Organization name is prostaglandin-endoperoxide synthase (Aoki and Narumiya, 2012; Wang D. et al., 2021). There are two COX isoforms: COX-1 (PTGS1) and COX-2 (PTGS1). COX-1 is constitutively expressed in most tissues, where it plays a role in maintaining tissue homeostasis by providing basal levels of prostaglandins (Wang B. et al., 2021). By contrast, COX-2 usually has limited expression in normal tissues, but it is highly inducible in response to IL-1β, TNFα and IFNγ, especially at sites of inflammation and during tumor progression (Hashemi Goradel et al., 2019). Both COX isoforms can transform arachidonic acid into prostaglandin G_2_ (PGG_2_) and, subsequently, into PGH_2_, which is finally converted into various prostaglandins *via* specific synthases **(** Figure 1 **)**. Prostaglandins then exert their actions by activating G-protein-coupled receptors on the cell surface, including the PGD_2_ receptors (DP1 and DP2), the PGE_2_ receptors (EP1, EP2, EP3, and EP4), the PGF_2α_ receptor (FP), the PGI_2_ receptor (IP) and the thromboxane A_2_ receptor (TP) (Funk, 2001). In some cases, nuclear receptors such as peroxisome proliferator-activated receptors (PPARs) can also be activated by certain prostaglandins or their metabolites (Wang and Dubois, 2010).

Constant exposure to proinflammatory stimulus, whether it is infectious or non-infectious, is responsible for the overexpression of COX-2 and development of chronic inflammation, which might lead to malignancies, such as hepatitis virus infection-related hepatocellular carcinoma (Lu et al., 2015), reflux esophagitis-related esophageal cancer (Yang et al., 2012) and inflammatory bowel disease-related (IBD) colorectal cancer (Ullman and Itzkowitz, 2011). Previous research demonstrated that COX-2 achieved cancer-promoting effects mainly by its downstream prostaglandins, which contributed to cancer initiation, progression and resistance to treatment (Hashemi Goradel et al., 2019; Hou et al., 2021). COX-2 can be expressed by cancer cells, cancer-associated fibroblasts (CAFs), tumor-associated macrophages (TAMs) and regulatory T (Treg) cells (Hashemi Goradel et al., 2019). The upregulated expression of COX-2 has been observed in numerous premalignant and malignant diseases, including colorectal adenoma, Barrett’s esophagus, colorectal cancer, gastric cancer, esophageal cancer, breast cancer, lung cancer, glioblastoma and so on (Wang and Dubois, 2006; Jiang et al., 2017; Wang D. et al., 2021). It is generally accepted that COX-2 contributes to carcinogenesis mainly *via* overproducing prostaglandins, especially PGE_2_.

**FIGURE 2:**
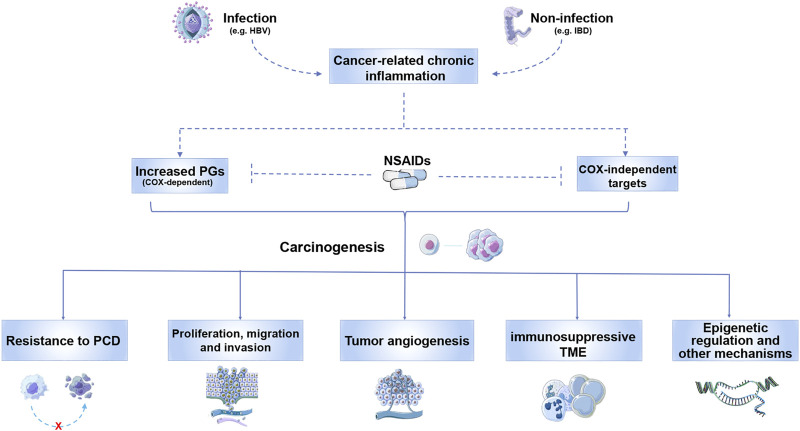
Tumor-promoting inflammation-related signals that can be targeted by NSAIDs. Both infectious and non-infectious chronic inflammation contributes to carcinogenesis *via* increasing PGs and activating COX-independent signals that can be suppressed by NSAIDs. These COX-dependent and independent pathways promote cancer development by inducing resistance to PCD, and facilitating proliferation, migration and invasion. Induction of tumor angiogenesis, immunosuppressive TME and other mechanisms are also achieved by NSAID-targeted signals. Abbreviations: HBV, hepatitis B virus; IBD, inflammatory bowel disease; PGs, prostaglandins; PCD, programmed cell death; TME, tumor microenvironment.

Besides COX enzymes, NSAIDs exert their function through COX-independent pathways as well. Several mechanisms have been proposed to demonstrate the tumor-promoting effects of NSAID-targeted signals, which are summarized as follows **(** Figure 2 **)**. Overproduction of PGE_2_ in tumor tissues usually results in resistance to apoptosis of cancer cell, as well as enhanced ability in proliferation, migration and invasion (Wang and Dubois, 2010; Lee et al., 2019; Cui et al., 2021). Besides, PGE_2_ promotes angiogenesis in cancer development (Zhang and Daaka, 2011; Xu et al., 2014). Generation of immunosuppressive tumor microenvironment (TME) by regulating tumor-infiltrating immune cells is also achieved by PGE_2_, which includes stimulating type-2 macrophage polarization, inducing T cell dysfunction and preventing tumor infiltration of dendritic cells or cytotoxic T lymphocytes (Ahmadi et al., 2008; Liu et al., 2012; Sun et al., 2022). The emergence of cancer stem cells (CSCs) is related to different PGE_2_-related signaling pathways as well (Li et al., 2012; Wang et al., 2015; Fang et al., 2017). Aspirin could promote apoptosis in CSCs in a COX-independent pathway (Chen et al., 2018). In addition, epigenetic regulation such as DNA methylation of tumor suppressive genes induced by PGE_2_ promotes cancer development (Xia et al., 2012; Wong et al., 2019). Except for PGE_2_, thromboxane A_2_, another COX-2 derived production, was reported to be related to enhanced tumor angiogenesis (Pradono et al., 2002). Whereas abundant evidences suggested contribution of COX-2 in various cancers, the role for COX-1 in cancer development remains much less discovered. Several studies showed that COX-1-dependent pathways was required for carcinogenesis, tumor growth and metastasis in certain malignancies as well (Daikoku et al., 2005; Li et al., 2014; Lucotti et al., 2019).

## Clinical outcomes of non-steroidal anti-inflammatory drugs in treating cancer-related inflammation

NSAIDs constitute a group of drugs with diverse chemical structure, which share a common mechanism of action by inhibiting COX activity and COX-independent pathways. Generally, all NSAIDs compete with arachidonate for the COX active site, which results in decreased production of prostaglandins. Now that COX enzymes and prostaglandins have been proved to be significantly related to cancer development, the application of NSAIDs becomes a promising strategy for the treatment of inflammation-driven cancers. The most commonly used NSAIDs and their clinical trials in chemoprevention or post-diagnosis use are listed in Table 1, and participants in chemoprevention trials were mostly with higher cancer risks.

**TABLE 1:** Classification, selectivity and anti-cancer clinical trials of classical NSAIDs.

Family/Class	Drug	Selectivity for COXs	Clinical trials in cancer prevention or treatment with NSAIDs	
			Chemoprevention	Post-diagnosis treatment
Salicylates	Aspirin	COX-1 and COX-2	Effective: esophageal adenocarcinoma, CRC Ineffective: breast cancer, lung cancer Unpublished results: melanoma, oral cancer	Effective: CRC, breast cancer
				Unpublished results: prostate cancer, melanoma
	Diflunisal	COX-2 selective	None	None
Acetic acid derivatives	Indomethacin	COX-1 and COX-2	None	Unpublished results: CRC, esophageal cancer, ovarian cancer, melanoma
	Sulindac	COX-1 and COX-2	Effective: colorectal adenoma Ineffective: lung cancer Unpublished results: oral cancer, melanoma	Effective: breast cancer, head and neck squamous cell carcinoma; Unpublished results: melanoma
	Diclofenac	COX-2 selective	None	Unpublished results: basal cell carcinoma
	Etodolac	COX-2 selective	None	None
Propionic acid derivatives	Ibuprofen	COX-1 and COX-2	None	None
	Flurbiprofen	COX-1 selective	None	None
	Naproxen	COX-1 and COX-2	Effective: CRC	None
Enolic acid derivatives	Piroxicam	COX-1 and COX-2	None	None
	Meloxicam	COX-2 selective	None	Effective: multiple myeloma
Diaryl heterocyclic compounds	Celecoxib	COX-2 selective	Effective: breast cancer, colorectal adenoma Ineffective: esophageal cancer, cervical cancer, oral cancer Unpublished results: NSCLC, CRC	Effective: ovarian cancer, cervical cancer, head and neck cancer, hepatocellular carcinoma, CRC Ineffective: breast cancer, NSCLC, esophageal cancer, prostate cancer, thyroid cancer, pancreatic cancer Unpublished results: bladder cancer, kidney cancer, oral cancer
	Rofecoxib	COX-2 selective	Effective: colorectal adenoma Unpublished results: prostate cancer	Effective: NSCLC Ineffective: CRC

*Information resource: https://clinicaltrials.gov Reported trials in this table are restricted to completed interventional studies. Abbreviations: COX, cyclooxygenase; NSAID, non-steroidal anti-inflammatory drug; CRC, colorectal cancer; NSCLC, non-small-cell lung cancer.

Chemoprevention strategies with NSAIDs have the potential to reduce incidence of several malignancies. Aspirin, one of the most widely used NSAIDs, has been identified as an effective cancer-preventive agent according to numerous epidemiological and clinical studies (Hou et al., 2021). A meta-analysis of 42 observational studies (99,769 cases) suggested an association between aspirin use and reduced incidence of breast cancer (Ma S. et al., 2021). Another meta-analysis based on individual-level data from nine cohort studies (2,600 cases) and 8 case-control studies (5,726 cases) identified a lower ovarian cancer risk associated with frequent aspirin use (Hurwitz et al., 2022). Likewise, the frequency of aspirin use was also emphasized by a meta-analysis in endometrial cancer (7 case-control and 11 cohort studies included, 14,766 cases in total), where the reduced cancer risk was closely related to the high-frequency of aspirin use instead of the duration of use (Wang et al., 2020). In addition to the frequency and duration of aspirin use, dose-effect relationship is a critical issue as well. A meta-analysis focusing on dose-effect relationship between aspirin and cancer risk revealed that high frequency or high dose use of aspirin might increase lung and prostate cancer risks, while low-dose of aspirin use could prevent colorectal cancer (Wang L. et al., 2021). Contrarily, a meta-analysis in hepatocellular carcinoma (HCC) (2 case-control and 16 cohort studies included) showed that the use of aspirin was associated with a lower risk of liver cancer, but not in a dose-dependent or a duration-dependent relationship (Wang et al., 2022). Interestingly, the meta-analysis in HCC concluded that aspirin had protective effects against HCC in patients with hepatitis B virus or hepatitis C virus infection (Wang et al., 2022), which was also supported by a nationwide study of patients with chronic viral hepatitis in Sweden (Simon et al., 2020). Moreover, according to a systematic review and meta-analysis of all observational studies on aspirin use and digestive-tract cancers up to March 2019, aspirin use was related to lower risks in various digestive malignancies, including colorectal cancer (45 studies), squamous-cell esophageal cancer (13 studies), adenocarcinoma of the esophagus and gastric cardia (10 studies), stomach cancer (14 studies), hepatobiliary cancer (5 studies) and pancreatic cancer (15 studies) (Bosetti et al., 2020). However, the findings of two large cohort studies didn’t support that aspirin use was associated with reduced pancreatic cancer risk, except in patients with diabetes (Khalaf et al., 2018). Despite the satisfactory chemoprevention effect of aspirin, it is noteworthy that prophylactic use of NSAIDs should be cautious with different populations. A randomized, double-blind, placebo-controlled trial (9,525 cases receiving aspirin and 9,589 cases receiving placebo) reported that older adults taking daily low-dose aspirin (100 mg) led to an increase in all-cause mortality, primarily due to cancer, and the follow-up data of this trial suggested that aspirin might accelerate the progression of cancer in older adults (McNeil et al., 2018; McNeil et al., 2021). COX-2 selective inhibitors like celecoxib and etodolac also have been proved to be efficient in chemoprevention for non-melanoma skin cancers and gastric cancer (Elmets et al., 2010; Yanaoka et al., 2010). Unlike generally accepted conclusion on the benefit of aspirin in preventing colorectal cancer, it is still questionable whether NSAIDs can reduce cancer risks in certain malignancies. For instance, lung cancer, one of the leading causes of cancer-related deaths, was proved to be of little association between its incidence and aspirin use according to different well-designed studies (Oh et al., 2011; Mc Menamin Ú et al., 2015; Loomans-Kropp et al., 2021). In hematologic malignancies, high use (≥4 days/week for ≥4 years) of acetaminophen was associated with increased incidence of myeloid neoplasms and non-Hodgkin’s lymphomas (Walter et al., 2011).

In addition to prophylactic use as chemoprevention strategies, NSAIDs can also improve the survival in patients who are already diagnosed with certain malignancies, which is supported by numerous observational studies and clinical trials. Post-diagnosis regular aspirin use was associated with reduced colorectal cancer-specific and overall mortality, especially in patients with positive PTGS2 (COX-2) expression and mutated PIK3CA tumors, reported by a meta-analysis (Li et al., 2015). Further studies revealed that, among colorectal cancer patients with low tumoral levels of PD- L1, survival benefit from post-diagnosis aspirin use was greater than in others (Domingo et al., 2013; Hamada et al., 2017). Another prospective cohort study of newly diagnosed biliary tract cancer (BTC) found that post-diagnosis aspirin use was associated with decreased BTC-specific mortality of different subtypes (Liao et al., 2021). In a cohort study of prostate cancer, similar results were observed in patients with high-risk cancers (≥T3 and/or Gleason score ≥8), where postdiagnosis daily aspirin use was associated with lower prostate cancer-specific mortality (Jacobs et al., 2014). In patients with esophageal, hepatobiliary and breast cancer, post-diagnosis use of aspirin was associated with increased survival as well (Fraser et al., 2014; Frouws et al., 2017).

Some clinical proofs supported that cancer patients undergoing radiotherapy or chemotherapy may benefit from additional use of NSAIDs. In prostate cancer patients treated with radiotherapy or radical prostatectomy, aspirin use was associated with a reduced risk of prostate cancer-specific mortality, especially in patients with high-risk disease (Choe et al., 2012). In pre-treated metastatic colorectal cancer patients receiving chemotherapy, aspirin improved overall survival significantly (Giampieri et al., 2017). In advanced non-small cell lung cancer (NSCLC) patients with COX-2 expression undergoing chemotherapy, who received celecoxib had better survival than that in non-users, according to a randomized clinical trial (Edelman et al., 2008). Aspirin could also decrease the proangiogenic effects of tamoxifen (a selective endocrine receptor modulator) in breast cancer patients, which suggested that antiplatelet or antiangiogenic therapy might improve the effectiveness of tamoxifen in breast cancer treatment (Holmes et al., 2008). In postmenopausal breast cancer patients treated with aromatase inhibitors, sulindac, a non-selective NSAID, reduced breast density, which is a risk factor for breast cancer, and the results implied that PGE_2_ inhibition by NSAIDs might be important for breast density change or collagen modulation during breast cancer development (Thompson et al., 2021). However, the benefit of NSAIDs in patients undergoing chemotherapy is not always satisfactory in certain cancer types or populations. In a randomized clinical trial of stage III colon cancer, additional celecoxib to standard adjuvant chemotherapy for 3 years did not significantly improve disease-free survival of the included patients, compared with patients receiving placebo (Meyerhardt et al., 2021). Similar results were reported by a trial in advanced non-small-cell lung cancer, where additional rofecoxib did not prolong the survival of patients receiving standard chemotherapy (Gridelli et al., 2007). In breast cancer patients receiving aromatase inhibitor treatment, short-term (≤18 months) celecoxib or low-dose aspirin use did not improve event-free survival or distant disease-free survival, and low-dose aspirin use even increased all-cause mortality (Strasser-Weippl et al., 2018).

The emergence of targeted therapies and immunotherapies has successfully prolonged overall survival for all kinds of cancer patients, and the use of NSAIDs along with targeted agents or immune checkpoint inhibitors seems promising. A retrospective analysis in epidermal growth factor receptor-mutant (EGFR) NSCLC patients suggested that concurrent aspirin use with osimertinib (EGFR inhibitor) was associated with prolonged progression-free survival (Liu et al., 2020), and a similar result was also reported in NSCLC (Han et al., 2020). In advanced hepatocellular carcinoma patients receiving sorafenib or regorafenib (multi-target tyrosine kinase inhibitors), concomitant use of aspirin improved their survival (Casadei-Gardini et al., 2021). A multicenter retrospective study showed that aspirin use was independently related to an increased objective response rate among 1012 cancer patients (52.2% NSCLC, 26% melanoma, 18.3% renal cell carcinoma and 3.6% others) treated with PD-1/PD-L1 inhibitors (Cortellini et al., 2020), and a meta-analysis reported that concurrent use of low-dose aspirin was associated with better progression-free survival in cancer patients receiving immune checkpoint inhibitors, including NSCLC (Zhang et al., 2021). Nevertheless, some studies revealed that NSAIDs did not benefit certain patients receiving targeted therapy or immunotherapy. In platinum refractory NSCLC patients, the combination of celecoxib and gefitinib (EGFR inhibitor) did not improve the response rate compared with gefitinib alone (Gadgeel et al., 2007). Immunotherapy with antibodies targeting cytotoxic T-lymphocyte-associated antigen 4 (CTLA-4) usually results in enterocolitis, and melanoma patients with anti-CTLA-4 enterocolitis took NSAIDs more frequently than patients without enterocolitis, which suggested that patients treated with anti-CTLA-4 were supposed to avoid NSAIDs (Marthey et al., 2016). In addition, concurrent use of immune checkpoint inhibitors and aspirin or NSAIDs did not improve disease control or survival in metastatic renal cell carcinoma patients, and use of NSAIDs was even associated with a higher risk of progression and death (Zhang et al., 2022).

## Drug metabolism, anti-cancer mechanisms and pharmacogenomics of non-steroidal anti-inflammatory drugs

### Pharmacogenomics in non-steroidal anti-inflammatory drug metabolism

The effectiveness of NSAIDs in cancer chemoprevention and post-diagnosis therapies varies in distinct populations and cancer types, primarily due to diverse genetic characteristics among different malignancies and individuals. The genetic variation of specific genes that are involved in drug metabolism or drug-induced signal transduction might affect the success of chemoprevention or treatment with NSAIDs (Scherer et al., 2014; Li et al., 2015). Taking account of such relevant pharmacogenomic differences has the potential to modify chemopreventive and therapeutic effects of NSAIDs.

Most NSAIDs are administered as active drugs, but some of them are prodrugs that require metabolic activation, such as sulindac. Several metabolic pathways are responsible for inactivation and elimination of NSAIDs, including oxidation (cytochrome P450 enzymes, CYP) and glucuronide conjugation (uridine-5′-diphosphate-glucuronosyl transferases, UGTs). The pharmacokinetic properties of NSAIDs vary among different individuals, partly because of their variance in NSAID metabolism-related genes, which affects plasma concentration and half-life of NSAIDs (Theken et al., 2020). CYP2C9 (cytochrome P450, family 2, subfamily C, polypeptide 9) is one of the most important enzymes for the oxidation of NSAIDs as well as other CYP enzymes, especially the CYP2C family (Theken et al., 2020). Besides, glucuronidation through UGTs is also an important pathway for NSAIDs clearance. UGT1A1, UGT1A6, UGT1A7, and UGT1A9 contribute most to aspirin glucuronidation, while the most important enzymes involved in non-aspirin NSAID glucuronidation are other members of the UGT family (Ulrich et al., 2006).

CYP2C enzymes and UGTs are highly polymorphic, and genetic variation of these genes plays a role in the inter-individual variability in NSAID elimination and efficacy. More than 60 variant alleles or multiple sub-alleles of CYP2C9 have been found, which can be categorized according to their functional status as follows: normal function (e.g., CYP2C9*1), decreased function (e.g., CYP2C9*2 and *5), and no function (e.g., CYP2C9*3 and *6) (Theken et al., 2020). Previous researches demonstrated that functional polymorphisms of CYP2C9 and UGT1A6 were related to modified effects of NSAIDs in the chemoprevention of colorectal adenoma and cancer (Bigler et al., 2001; Chan et al., 2005; Samowitz et al., 2006; Chan et al., 2009; Yamazaki et al., 2021). In addition, other functionally relevant polymorphisms of UTGs were also associated with modification of NSAID effectiveness on colorectal cancer risk (Angstadt et al., 2014; Scherer et al., 2014). These inspiring findings emphasize the need for further pharmacogenomic researches to identify individuals that might benefit from NSAIDs in cancer chemoprevention.

### Anti-cancer mechanisms and pharmacogenomics of non-steroidal anti-inflammatory drugs

According to classical theories, NSAIDs exert their anti-cancer effects mainly based on COX-dependent mechanisms *via* inhibiting COX-2 activity and prostaglandin production. Nevertheless, emerging evidences presented some novel targets in COX-independent anti-tumor effects, where NSAIDs directly interacted with proteins other than COX enzymes. COX-independent molecular targets are summarized in Table 2. Pharmacogenetic studies have demonstrated that genetic variation is one of the leading causes of variability in drug response. Among different types of genetic variations that affect inter-individual drug response, single nucleotide polymorphisms (SNPs) play a critical role due to their occurrence frequency of >1% in the human population (Gholamian Dehkordi et al., 2021). Because different NSAIDs have various molecular targets, including the most classical COX enzymes and COX-independent molecules, genetic variations in these genes and their closely related upstream or downstream genes can be tremendous. Therefore, identification of certain determining pharmacogenomic features is critical for predicting drug response and select eligible patients to receive NSAIDs along with standard anti-cancer treatment. For instance, aspirin use was associated with reduced rate of colorectal cancer recurrence in patients with PIK3CA-mutant tumors compared with patients with PIK3CA wild-type tumors (Domingo et al., 2013). Therefore, PIK3CA mutations could be regarded as an effective pharmacogenomic feature in predicting aspirin effectiveness in colorectal cancer patients.

**TABLE 2:** COX-independent molecular targets of NSAIDs.

Targets		Effects of NSAIDs	References
Nuclear receptors	PPAR-γ	Stimulation of PPAR-γ; inhibition of cancer cell growth and enhancement of cell migration	Wick et al. (2002); Babbar et al. (2003); Kato et al. (2011)
	PPAR-δ	Inhibition of PPAR-δ; induction of cancer cell apoptosis	He et al. (1999); Liou et al. (2007)
	RXRα	Inhibition of RXRα, induction of cancer cell apoptosis	Kolluri et al. (2005); Zhou et al. (2010)
Transcription factors	NF-κB	Inhibition of NF-κB; suppression of cancer proliferation, angiogenesis and metastasis	Shishodia and Aggarwal, (2004); Feng et al. (2007); Liao et al. (2015)
	AP-1	Inhibition of AP-1; suppression of malignant transformation induced by tumor promotor	Dong et al. (1997); Liu et al. (2003)
	Sp1	Inhibition of Sp1; suppression of tumor angiogenesis	Wei et al. (2004)
Kinases	AMPK/mTOR	Activation of AMPK and inhibition of mTOR; induction of autophagy in cancer cells	Din et al. (2012); Hawley et al. (2012)
	PDPK-1	Inhibition of PDPK-1; suppression of tumor proliferation and induction of apoptosis	Arico et al. (2002); Kulp et al. (2004)
Others	PDE5	Inhibition of PDE5; suppression of tumor cell growth and induction of apoptosis	Tinsley et al. (2009); Tinsley et al. (2010)
	SERCA	Inhibition of SERCA; induction of cancer cell apoptosis	Johnson et al. (2002); White et al. (2013)
	Histone acetyltransferase p300	Interaction with p300; induction of CSC apoptosis and suppression of tumor progression	Chen et al. (2018)

Accumulating proofs suggested that NSAIDs were capable of suppressing proliferation, migration and invasion in cancer cells and promoting their programmed cell death. Selecting patients with potential benefits based on their pharmacogenomic characteristics was also supported by some evidences. Various NSAIDs suppressed NF-κB-regulated COX-2 expression in a dose-dependent manner and inhibited the proliferation of tumor cells (Takada et al., 2004). Inhibition of NF-κB pathway induced by aspirin suppressed the growth, migration and metastasis of osteosarcoma (Liao et al., 2015). NF-κB polymorphisms had an impact on cancer risks, and carriers of specific NF-κB variants might benefit from NSAIDs in cancer chemoprevention (Chang et al., 2009; Seufert et al., 2013). In addition to NF-κB, mammalian target of rapamycin (mTOR) pathways can be affected by NSAIDs as well. Aspirin suppressed mTORC1 signaling and the PI3K/AKT, MAPK/ERK pathways, and it showed synergetic anti-cancer efficacy in combination with sorafenib in hepatocarcinoma cells (Sun et al., 2017). By inhibiting AKT/mTOR signaling, aspirin also promoted RSL3-induced ferroptosis in PIK3CA-mutant colorectal cancer cells (Chen et al., 2022). In hepatocellular carcinoma, celecoxib acted synergistically with chemotherapeutic drugs in promoting apoptosis, and celecoxib induced COX-2 inhibition in different apoptotic pathways, including stimulating death receptor signaling, activation of caspases and mitochondrial apoptosis pathway (Kern et al., 2006). Combined use of celecoxib and erlotinib (EGFR inhibitor) also suppressed prostaglandin signaling and promoted apoptosis of intestinal tumors *in vivo* (Buchanan et al., 2007). Aspirin even triggered disruption of the chromosomal architecture of the COX-2 locus in lung cancer cells during radiation treatment and increased the level of apoptosis (Sun et al., 2018). In lymphoma B cells, celecoxib enhanced the apoptotic activity of TRAIL (TNF-related apoptosis-inducing ligand) through COX-2-independent effects *via* decelerating the cell cycle and inhibiting expression of survival proteins, like Mcl-1 (Gallouet et al., 2014). Similar targets were verified in colon cancer cells, where combined use of aspirin with sorafenib suppressed proliferation by targeting the anti-apoptotic proteins FLIP and Mcl-1 and sensitized cancer cells to TRAIL (Pennarun et al., 2013). Sulindac could also induce apoptosis by binding to retinoid X receptor-alpha rather than COX, which inhibited TNFα induced PI3K/AKT signaling and activated the death receptor-mediated apoptotic pathway (Zhou et al., 2010). The combination of aspirin and osimertinib inhibited AKT/FOXO3a signaling component phosphorylation and increased Bim expression in osimertinib-resistant NSCLC cells and promoted Bim-dependent apoptosis, which decreased tumor growth *in vivo* (Han et al., 2020).

Inhibition of tumor angiogenesis is also a significant function of NSAIDs. PGE_2_-EP3 signaling induced tumor metastasis and angiogenesis by upregulation of matrix metalloproteinase-9 (MMP-9), which could be suppressed by NSAIDs (Amano et al., 2009). Another study proved that PGE_2_ biosynthesis was dependent on COX-1 rather than COX-2 in endothelial cells, which could be blocked by aspirin *in vivo* (Salvado et al., 2013). Overexpression of COX-2 stimulated the expression of angiogenic-related genes in breast cancer cells isolated from COX-2 transgenic mice, and treatment with celecoxib suppressed tumor growth and micro-vessel density (Chang et al., 2004). Vascular endothelial growth factors (VEGF) have been identified as major mediators of tumor angiogenesis, and aspirin decreased serum level of VEGF and suppressed the pro-angiogenic effects of tamoxifen in breast cancer patients, where interindividual variability was noted by the researchers (Holmes et al., 2008). Genetic variations in VEGF-A and its receptors 1 (FLT1) and 2 (KDR) were proved to be associated with colon cancer survival, and the association could be modified by NSAID use, which indicated that cancer patients with specific SNPs in these genes could benefit more from NSAIDs (Slattery et al., 2014). In patients with cervical intraepithelial neoplasia 3 (CIN 3), serum VEGF levels were helpful to identify patients who may benefit from celecoxib, which provided novel strategies to cervical cancer chemoprevention (Rader et al., 2017). These results implied the existence of undiscovered pharmacogenomic features related to anti-angiogenesis effects of NSAIDs in cancer treatment.

*Cancer* stem cells (CSCs) play an important role in cancer recurrence, metastasis and resistance to drugs, and NSAIDs can reduce cancer stem cells according to some researches. In colorectal cancer, NSAIDs (indomethacin, sulindac, aspirin and celecoxib) could inhibit the formation of CSCs and reduce chemotherapy-induced CSCs *via* inhibiting COX-2 and NOTCH/HES1, and activating PPARγ (Moon et al., 2014). Chemotherapeutic drugs could generate CSCs through an NFκB-IL6-dependent inflammatory environment and result in multidrug resistance in breast cancer, but treatment with aspirin was able to disturb the nuclear translocation of NF-κB in CSCs and improve sensitivity to chemotherapy (Saha et al., 2016). Aspirin could eliminate colorectal CSCs in a COX-independent pathway, where aspirin directly interacted with histone acetyltransferase p300, promoted H3K9 acetylation, activated FasL expression, and resulted in apoptosis in CSCs (Chen et al., 2018).

NSAIDs can also affect the epigenetic regulation of certain genetic loci, which results in anti-cancer effects. Aspirin could reduce histone demethylase (KDM6A/B) expression and suppress the expression of inflammation-related stemness genes (especially ICAM3), and inhibit tumor growth and metastasis (Zhang et al., 2020). A population-based study revealed that aspirin users with unmethylated promotor of BRCA1 and global hypermethylation of long interspersed elements-1 (LINE-1) had lower breast cancer-specific mortality (Wang et al., 2019). This study provided important pharmacogenetic evidence, which implied that epigenetic features of specific susceptibility genes should be taken into consideration before NSAID use.

Evidences showed that the regulation of immune cells in TME was achieved by NSAIDs as well. A prospective cohort study showed that regular aspirin use was related to a lower risk of colorectal carcinomas with low concentrations of tumor-infiltrating lymphocytes (TILs), which implied that aspirin contributed to the increased TILs in tumor tissues (Cao et al., 2016). Inhibition of the COX-2/PGE_2_/EP4 axis increased tumor-infiltrating immune cells in the microenvironment and restored sensitivity of drug-resistant tumor to pembrolizumab (Pi et al., 2022). The risk of developing breast cancer can be increased by radiotherapy for existing malignancies, post-irradiation use of low-dose aspirin for 6 months in mice could prevent the establishment of an immunosuppressive tumor microenvironment, which was characterized by enriched proinflammatory factors and abundant myeloid cells, and aspirin intervention signiflcantly decreased COX-2 and TGFβ intensity in tumors from irradiated hosts (Ma L. et al., 2021).

In addition to the well-known focus on cancer proliferation, programmed cell death, angiogenesis, stemness, epigenetic regulation and immune regulation, other mechanical and clinical researches revealed some promising pharmacogenomic features as well. A large-scale case-control study showed that NSAID use was associated with reduced risk of colorectal cancer, and the association varied according to genetic variation at two SNPs at chromosomes 12 and 15 (Nan et al., 2015). Tumor repopulation is a major cause of radiotherapy failure, and pancreatic cancer repopulation upon radiation was suppressed by aspirin *in vitro* and *in vivo via* inhibiting dying tumor cells from releasing exosomes and PGE_2_, which were critical for the survival and proliferation of tumor repopulation cells (Jiang et al., 2020). This study suggested that pancreatic cancer patients undergoing radiotherapy might benefit from combined use of aspirin. A study proved that tumor sensitivity to radiotherapy was enhanced by four tested NSAIDs (diclofenac, indomethacin, piroxicam and NS-398) *via* increasing tumor oxygenation, which was primarily mediated by an effect on mitochondrial respiration (Crokart et al., 2005). In lung cancer, aspirin caused disruption of the chromosomal architecture in the COX-2 locus and reduced its production in cell lines, which enhanced radiosensitivity of lung cancer cells (Sun et al., 2018).

Considerable amounts of studies demonstrated the effectiveness of NSAIDs in chemoprevention and post cancer-diagnosis use in certain cancer types, but there is still requirement for more researches in comprehensively clarifying the underlying anti-cancer mechanisms of NSAIDs, as well as exploring more pharmacogenomic features to guide personalized chemoprevention and treatment.

## Conclusion and future directions

Chronic inflammation results in upregulation of proinflammatory molecules, recruitment of inflammatory cells, genetic and epigenetic alterations in normal cells, thus initiating carcinogenesis and cancer development. NSAIDs are capable of suppressing various aberrantly activated genes during inflammation and cancer progression, including the most classical COX enzymes and other COX-independent pro-cancer genes. Numerous epidemiological, clinical and mechanical researches revealed the effectiveness of NSAIDs in chemoprevention and post cancer-diagnosis treatment in both solid tumors and hematological malignancies. However, NSAIDs do not benefit every individual with cancer risk, particularly because of their genetic variations in NSAID-related genes. Detecting such pharmacogenomic features among normal people or cancer patients makes it possible to select individuals who might benefit from NSAIDs in chemoprevention or anti-cancer treatment.

Besides pharmacogenomic features, other factors might have an impact on the effectiveness or toxicity of NSAIDs as well. The dose, duration and frequency of NSAID use is a critical issue. An average daily dose of 100 mg of coated aspirin have favorable preventive effects on cancer, and cancer-specific survival benefit is achieved with aspirin doses as low as 80 mg daily (Chia et al., 2012; Lotrionte et al., 2016). The effective dose and frequency of celecoxib in chemoprevention and treatment varied among different populations, and some recommended doses and frequencies are listed as follows: 400 mg daily for preventing recurrence of breast cancer and colorectal adenoma, 600 mg bid for NSCLC patients receiving erlotinib and 16 mg/kg/day for children with colorectal cancer risk (Reckamp et al., 2006; Lynch et al., 2010; Saxena et al., 2020). Researches focusing on relationship between duration and effectiveness of NSAID use remains limited. Consistent aspirin use over 6 years reduced colorectal cancer risk among men (Chan et al., 2008). Aspirin use over 10 years significantly reduced HCC incidence while the use for 5–10 years only achieved marginal reduction (Fujiwara et al., 2019). Pharmacokinetic interaction with other anti-cancer drugs also affected the effectiveness of NSAIDs. Ibuprofen co-administered with pemetrexed suppressed the clearance of pemetrexed and increased its maximum plasma concentration (Sweeney et al., 2006). Coadministration of celecoxib and capecitabine increased celecoxib exposure in patients, which suggested the importance of close monitoring of cancer patients receiving NSAIDs with a narrow therapeutic index (Ramírez et al., 2019). In addition, selective COX-2 inhibitors such as celecoxib have been associated with great risk of adverse cardiovascular effects, and aspirin use was associated with a higher risk of major bleeding in individuals without cardiovascular disease (Zheng and Roddick, 2019; Schjerning et al., 2020). Such NSAID-related adverse events must be considered before use.

Novel technologies such as liquid biopsy and next generation sequencing have enabled the quick and sensitive detection of pharmacogenomic features among cancer patients (Bignucolo et al., 2017). Early detection and real-time monitoring of NSAID-related pharmacogenomic features could help identify individuals with specific genomic characteristics related to NSAID sensitivity and allow precise selection of patients, thus achieving successful personalized chemoprevention and treatment for cancer.

## Abbreviations

COX, cyclooxygenase; NSAID, non-steroidal anti-inflammatory drug; PPAR, peroxisome proliferator-activated receptors; RXRα, retinoid X receptor α; NF-κB, Nuclear factor-kappa B; AP-1, activator protein 1; SP-1, Transcription factor Sp1; AMPK, adenosine monophosphate-activated protein kinase; mTOR, mechanistic target of rapamycin; PDPK-1, 3-phosphoinositide-dependent protein kinase 1; PDE5, phosphodiesterase 5; SERCA, sarcoendoplasmic/reticulum Ca^2+^ATPase; CSC, cancer stem-like cells.
